# Variations in genome-wide RNAi screens: lessons from influenza research

**DOI:** 10.1186/s13336-015-0017-5

**Published:** 2015-03-03

**Authors:** Yu-Chi Chou, Michael MC Lai, Yi-Chen Wu, Nai-Chi Hsu, King-Song Jeng, Wen-Chi Su

**Affiliations:** National RNAi Core Facility Platform, Academia Sinica, Taipei, 11529 Taiwan; Institute of Molecular Biology, Academia Sinica, Taipei, 11529 Taiwan; Research Center for Emerging Viruses, China Medical University Hospital, Room 602, 6 F, Cancer Center Building, No. 6, Hsueh-Shih Road, Taichung, 40402 Taiwan; China Medical University, Room 602, 6 F, Cancer Center Building, No. 6, Hsueh-Shih Road, Taichung, 40402 Taiwan; Center of Infectious Disease and Signaling Research, National Cheng Kung University, Tainan, 70101 Taiwan

**Keywords:** RNA interference (RNAi), Genome-wide screen, Arrayed siRNA screen, Pooled shRNA screen, Influenza virus, Lentivirus

## Abstract

Genome-wide RNA interference (RNAi) screening is an emerging and powerful technique for genetic screens, which can be divided into arrayed RNAi screen and pooled RNAi screen/selection based on different screening strategies. To date, several genome-wide RNAi screens have been successfully performed to identify host factors essential for influenza virus replication. However, the host factors identified by different research groups are not always consistent. Taking influenza virus screens as an example, we found that a number of screening parameters may directly or indirectly influence the primary hits identified by the screens. This review highlights the differences among the published genome-wide screening approaches and offers recommendations for performing a good pooled shRNA screen/selection.

## Introduction

RNA interference (RNAi) is a revolutionary technique for studying the biological functions of a particular gene by silencing its gene expression that can be applied in mammalian systems. The manipulation of gene expression of any particular gene by RNAi provides insight into the genetic networks related to the target gene. Thus far, the main approaches for RNAi screening can be divided into two screening formats, arrayed screen and pooled selection/screen. For arrayed screen, each RNAi reagent is assigned to a unique well in a microplate, and a variety of cell-based assays are performed in the microplate format. After the assay, cells with significant changes can be identified and the related RNAi reagents can be traced by using a database. For pooled selection/screen, all of the RNAi reagents are pooled together and added randomly to cells. There are two strategies for pooled selection: positive selection, which only detects surviving cells and does not require an untreated control, and negative selection, which includes an untreated control for comparison to allow the detection of RNAi reagents that make the cells resistant or sensitive to the selective reagent. The significant RNAi reagents are subsequently deconvoluted by using Next Generation Sequencing (NGS) or barcode microarray.

Influenza virus causes annual epidemics and recurring pandemics which potentially threaten public health and the global economy. Influenza A viruses (IAVs) are enveloped RNA viruses with single-stranded, negative-sense viral RNAs encoding 11 viral proteins [1]. Two of the viral proteins, neuraminidase (NA) and matrix protein 2 (M2), are the targets of the currently-used antiviral drugs. However, the high error rate of the viral RNA-dependent RNA polymerase (RdRp) leads to rapid changes in these two proteins and generation of drug-resistant influenza viruses. It has been widely recognized that the replication of influenza virus relies on host factors and cellular machinery to complete its life cycle. Accordingly, identification of the host factors involved in viral replication is of interest to understand the mechanisms of the virus replication cycle more comprehensively and to find new targets for the development of antiviral compounds. Several genome-wide RNAi screens (summarized in Table 1) have been conducted for influenza virus to identify host factors required for influenza virus replication and provide robust information regarding the screening method. Here, we review these studies of genome-wide RNAi screens and compare the different screening approaches and the screening results obtained from these screens.

**Table 1:** **Genome-wide RNAi screens in influenza virus research (2008–2013)**

	**Hao et al. (2008) [** 2 **]**	**Brass et al. (2009) [** 3 **]**	**Shapira et al. (2009) [** 4 **]**	**Kŏnig et al. (2010) [** 5 **]**	**Karlas et al. (2010) [** 6 **]**	**Su et al. (2013) [** 7 **]**	**Tran et al. (2013) [** 9 **]**
No. of genes screened	13,071	17,877	1,745 (pre-selected by yeast −2-hybrid and microarray)	19,628	22,843	16,368	21,415
Primary screen (Source of RNAi library)	Arrayed dsRNA screen (Ambion)	Arrayed siRNA screen (Dharmacon)	Arrayed siRNA screen (Dharmacon)	Arrayed siRNA screen (Qiagen)	Arrayed siRNA screen (Qiagen)	Pooled shRNA screen (TRC)	Pooled shRNA screen (Thermo)
Delivery method	Bathing	Transfection	Transfection	Transfection	Transfection	Lentiviral	Lentiviral
Cell used for screen (Origin)	DL1 cells (Drosophila)	U2OS cells (Human)	HBEC cells (Human)	A549 cells (Human)	A549/293 T cells (Human)	A549 cells (Human)	A549 cells (Human)
Virus strain	Recombinant A/WSN/33 (WSN; H1N1) virus possessing VSV-G and renilla Luciferase gene	A/Puerto Rico/8/34 (PR8; H1N1)	A/Puerto Rico/8/34 (PR8; H1N1)	Recombinant A/WSN/33 (WSN; H1N1) virus possessing renilla Luciferase gene	A/WSN/33 (WSN; H1N1)	A/WSN/33 (WSN; H1N1)	A/NY/55/2004 (NY55; H3N2)
Detection Method & Screen parameter	Reporter (Luciferase activity)	HA expression on cell surface	Reporter (Luciferase activity)	Reporter (Luciferase activity)	NP staining/Inducible influenza-virus-specific luciferase	Deep sequencing of shRNA which pertubed the cytopahic effect by influenza virus	Deep sequencing of shRNA which pertubed the cytopahic effect by influenza virus
Length of RNAi treament (days)	2 days	3 days	3 days	2 days	2 days	Over 2 weeks	over 2 weeks
Analysis time point after influenza virus infection	24 hrs	12 hrs	48 hrs	12, 24, 26 hrs	24 hrs	4 weeks	72 hrs
Methods to reduce off-target effects	Biological replicates/alternate control dsRNA	Biological replicates/ two or more siRNAs targeting to one gene	NA	Biological replicates/ two or more siRNAs targeting to one gene	Two or more siRNAs targeting to one gene	Two or more siRNAs targeting to one gene	Biological replicates
No. of candidate genes (Primary screen)	110	133	616	295	287	110	138
Steps in the viral life cycle covered by the primary screen	Uncoating, nuclear import, transcription, translation	Entry, uncoating, nuclear import, transcription, translation, HA trafficking	Entry, uncoating, nuclear import, transcription, translation, virus assembly, budding	Uncoating, nuclear import, transcription, translation	Entry, uncoating, nuclear import, transcription, translation, virus assembly, budding	Entry, uncoating, nuclear import, transcription, translation, virus assembly, budding	Entry, uncoating, nuclear import, transcription, translation, virus assembly, budding
Secondary screen for validation	No	No	No	Wild-type A/WSN/33 (WSN; H1N1) virus	A/WSN/33 and A/Hamburg/04/2009	High-content screening for NP staining	Viability of infected cells after siRNA transfection
No. of candidate genes (Secondary screens)	NA	NA	NA	219	168	38	~2/3 of 138 candidate genes
Steps in the viral life cycle covered by the secondary screen	NA	NA	NA	Entry, uncoating, nuclear import, transcription, translation, virus assembly, budding	Entry, uncoating, nuclear import, transcription, translation, virus assembly, budding	Entry, uncoating, nuclear import, transcription, translation	NA

### Genome-wide arrayed RNAi screens for the identification of host factors affecting influenza virus replication

The first genome-wide RNAi screen for the identification of host factors required for influenza virus replication was performed in a Drosophila cell line by Hao et al. [2] in 2008, when the RNAi-based screening was not yet well established in the mammalian system. To bypass the entry block of wild-type influenza virus into Drosophila cells, the authors generated a recombinant A/WSN/33 virus possessing vesicular stomatitis virus glycoprotein G (VSV-G) as a viral envelope protein to mediate its entry into target cells and carrying renilla luciferase gene as a reporter to assess the efficiency of virus RNA replication. A dsRNA library targeting 13,071 Drosophila genes was arrayed into 384-well microplates and applied for screening the host factors which modulate influenza virus RNA replication. A total of 110 candidate genes were identified by this screen, three of which (corresponding human orthologs; ATP6V0D1, COX6A1 and NXF1) had been validated to play critical roles in the replication of H5N1 and H1N1 influenza A viruses in mammalian cells. This study set up a framework for genome-wide RNAi screening to identify unknown host factors required for influenza virus replication.

In 2009, Brass et al. [3] performed a genome-wide arrayed RNAi screen (17,877 genes; Dharmacon) in human U2OS cells and identified 133 host factors required for influenza virus replication as evidenced by the alteration of HA expression on the cell surface. In this study, the candidate genes were selected based on the criteria that the gene was knocked down by at least 2 unique siRNAs to minimize the probability of off-target effects. Three interferon-inducible transmembrane proteins (IFITM1, IFITM2 and IFITM3) were identified to be antiviral restriction factors during virus infection. Shapira et al. [4] used a pre-selected gene list (1,745 genes) based on integrated yeast two-hybrid and microarray data, which were obtained from influenza-related studies, to identify cellular modifiers of influenza virus replication in human HBEC cells. The authors identified 616 host factors that were proposed to be involved in modulation of virus replication and/or interferon production.

König et al. [5] and Karlas et al. [6] also utilized a modified genome-wide RNAi screening approach by using an arrayed siRNA library (Qiagen). König et al. generated recombinant influenza virus possessing renilla luciferase gene for the primary screen, and identified 295 host factors out of the 19,628 genes in mammalian cells. Among the 295 candidate genes, 219 host factors were further confirmed to be essential for efficient replication of the wild-type influenza virus (A/WSN/33) in human A549 cells. On the other hand, Karlas et al. took a two-step strategy to survey host factors involved in influenza virus replication. In the first step, A549 cells were transfected with siRNA, and then infected with wild-type influenza virus (A/WSN/33). In the second step, the virus supernatants were collected and used to challenge HEK293T carrying an influenza virus-driven luciferase reporter for primary screen. After screening of an arrayed RNAi library (22,843 genes), a total of 287 genes were identified as primary hits. The secondary screen was performed by using A/WSN/33 and A/Hamburg/04/2009 independently, and 168 out of the 287 primary hits were validated to modulate influenza virus replication. Notably, 72 of them were common host factors affecting the replication of both influenza virus strains.

### Genome-wide pooled RNAi screens applied to identify host factors required for influenza virus replication

Rather than performing the arrayed RNAi screens (dsRNA or siRNA) as mentioned above, we established a genome-wide pooled RNAi screen (lentiviral shRNA expression system) based on a positive selection strategy to identify host factors required for influenza virus replication [7]. Under positive (survival) selection, cells with gene knockdown that provide resistance to influenza virus-induced cell death were selected and enriched in the population during pooled RNAi selection. The increase of the resistant cells in the population makes identification of the contents of shRNAs possible. In brief, A549 cells were infected with the pooled lentiviruses (multiplicity of infection; MOI = 0.3) generated from a genome-wide RNAi library from the RNAi consortium (TRC) [8], and subsequently challenged with influenza virus (A/WSN/33) at a cytotoxic dose. The surviving A549 cells were collected and subjected to deep sequencing to identify the embedded shRNA(s) that silenced host genes and protected cells from influenza virus-induced cell death. Ideally, host genes identified by this positive selection should be the essential factors supporting influenza virus replication. Out of the 16,368 genes screened, a total of 110 host genes targeted by at least two unique shRNA per gene were identified as our primary hits. The expression levels of these hits in A549 cells were further confirmed by EST or microarray analyses. We next carried out a high content image-based screen as a secondary screen and found that at least 38 candidate genes were involved in the early stages of the virus life cycle. Among them, E3 ubiquitin ligase Itch was proven to be an essential host factor for influenza virus “uncoating” [9]. Thus, we concluded that the genome-wide pooled RNAi screen via positive selection is a useful RNAi screen method for exploring host factors of lytic viruses.

Simultaneously, Tran et al. [9] also used a similar screening approach, by using a higher influenza virus dose with a shorter infection time, to explore specific host genes required for influenza virus-induced cell death. A genome-wide pooled RNAi library (Thermo Scientific Open Biosystems; also known as an RNAi library from the Hannon and Elledge Lab) targeting 21,415 host genes were screened and a total of 138 host genes were identified to be involved in influenza virus (A/NY/55/2004) replication. The authors validated these hits and identified APRIL, TWE-PRIL and USP47 as essential host factors for supporting influenza viral replication.

### Comparing host factors involved in influenza virus replication among different genome-wide RNAi screens

A total of 1,362 host factors were identified as potential cellular mediators in relation to influenza virus replication by the seven aforementioned RNAi screens. More than 90% of the hits (1,229 genes) were identified by only one of the RNAi screens. 113 overlapping host genes were identified in two independent RNAi screens, 14 overlapping genes (ARCN1, ATP6V0B, ATP6V1B2, COPB2, MAPK13, NUP98, PGD, PRPF8, RAB5A, RNF44, RPS10, RPS16, STARD5 and TRIM21) were identified in three RNAi screens, and 6 overlapping genes (ATP6AP1, ATP6V0C, ATP6V0D1, COPA, COPG and NXF1) were identified in four individual screens (Table 2). However, no host factors were commonly identified in all seven screens. Among the overlapping hits, many mapped to modulation of specific steps of the influenza virus life cycle, such as endocytosis (e.g., RAB5A) [10]; COPI vesicular transport (e.g., ARCN1, COPA, COPB2 and COPG) [11]; V-type ATPase proton transport (e.g., ATP6AP1, ATP6V0B, ATP6V0C, ATP6V0D1 and ATP6V1B2) [12]; nuclear import (e.g., NUP98) [13]; pre-mRNA splicing (e.g., PRPF8); nuclear export (e.g., NXF1) [14]; and protein translation (e.g., RPS10 and RPS16). Therefore, genome-wide RNAi screens apparently constitute a reliable approach to identify authentic host factors involved in influenza virus replication, but these screens also possess some intrinsic difficulties that may hinder the design of an ideal screening procedure.

**Table 2:** **Gene symbols and numbers of overlapping hits identified by RNAi screens**

**Gene symbol**	**Number**	**Gene symbol**	**Number**	**Gene symbol**	**Number**
ATP6AP1	4	CLOCK	2	PHF2	2
ATP6V0C	4	COPB1	2	PHF3	2
ATP6V0D1	4	DAPK2	2	PLK3	2
COPA	4	DCLRE1A	2	PLXNA2	2
COPG	4	DCLRE1C	2	PNMA1	2
NXF1	4	DHCR24	2	POLD3	2
ARNC1	3	DLG5	2	PPAN	2
ATP6V0B	3	DPF2	2	PPARA	2
ATP6V1B2	3	EIF2AK2	2	PPP1R14D	2
COPB2	3	EIF4A2	2	PPP2R2D	2
MAPK13	3	EPHA7	2	PQLC1	2
NUP98	3	EPHB2	2	PRKACA	2
PGD	3	ERCC4	2	PSENEN	2
PRPF8	3	FAM38A	2	PSMD11	2
RAB5A	3	FAU	2	PSMD14	2
RNF44	3	FGFR2	2	PTPN6	2
RPS10	3	FLNC	2	PTPRN	2
RPS16	3	HIST1H2AC	2	PTS	2
STARD5	3	HRAS	2	RAB10	2
TRIM21	3	IFIT5	2	RACGAP1	2
ABCC10	2	IGSF1	2	RIOK3	2
APOBEC3G	2	IKBKE	2	RP2	2
ARTN	2	IL17RA	2	RPL13A	2
ATF1	2	IL1A	2	RPS14	2
ATP6AP2	2	IRF2	2	RPS20	2
ATP6V1A	2	ISG15	2	RPS4X	2
B2M	2	IVNS1ABP	2	RPS5	2
BPTF	2	JUN	2	RRP1B	2
BUB3	2	KPNB1	2	RUNX1	2
BZRAP1	2	KRTCAP2	2	SAMHD1	2
C14orf109	2	MADD	2	SELPLG	2
C21orf33	2	MAP2K3	2	SF3A1	2
C5orf38	2	MAP3K12	2	SF3B1	2
C6orf62	2	MDM2	2	SIGMAR1	2
CALCOCO2	2	MED6	2	SLC1A3	2
CAMK2B	2	MFAP1	2	SNRP70	2
CCL13	2	MPG	2	SUPT6H	2
CD81	2	MYC	2	TCF7L2	2
CDC42BPA	2	NHP2L1	2	TNFRSF18	2
CDKN2AIP	2	NR4A2	2	TNK2	2
CFLAR	2	OSMR	2	TOPORS	2
CLEC2B	2	P2RY12	2	TRIM28	2
CLIC4	2	PAGE5	2	VCP	2
CLK1	2	PDGFRA	2	WTAP	2
				ZNF154	2

### Factors that lead to successful identification of specific host factors by RNAi screens

Only a limited number of overlapping host factors were identified among these RNAi screens, suggesting that the parameters used in the different RNAi screening protocols likely affect the screening results. Relevant parameters may include: (i) characteristics of influenza virus (virus strain, virus quality, viral subtype, prototype or recombinant virus) and host cell line (cell type, cell quality, genetic profile); (ii) features of the RNAi library screened, particularly the knockdown efficiency of the RNAi resources, and the quality of the RNAi library; (iii) screening method/time points for analysis; and (iv) hit-selection criteria and evaluation of proper controls, e.g., at least two siRNAs or shRNAs targeting to the same gene, and z-score of RNAi screen. Screens that used similar screening resources or approaches showed a higher degree of concordance. For example, Brass et al. and Shapira et al. used the same source of siRNA library (Dharmacon) and influenza virus strain (A/Puerto Rico/8/34) for arrayed siRNA screens, and in their lists of primary gene hits there were 16 overlapping host factors. Similarly, König et al. and Karlas et al. used the same siRNA library (Qiagen) and influenza virus strain (A/WSN/33) for arrayed siRNA screens and identified 25 overlapping host factors (Table 3). Moreover, the steps of influenza virus replication covered by RNAi screening may determine the populations of host factors identified in the primary screens. For example, Shapira et al. and Tran et al. performed RNAi screens aiming at the entire viral life cycle; 24 overlapping host factors were identified in their primary hits (Table 3). It is also noteworthy that both groups analyzed the results at similar time points (2–3 days) after influenza virus infection. On the other hand, our screen strategy was based on cell survival and analyzed the hits at a later time point (4 weeks) after influenza virus infection [7]. Under such stringent selection conditions, some host factors targeted by effective shRNAs, such as factors for cell survival or cell proliferation, are likely completely excluded from our primary hits after long-term selection; as a result, our hit list includes fewer overlaps with those identified by other RNAi screens. Nonetheless, the hits identified are more likely to represent better drug targets as compared to those identified by the arrayed siRNA screens (see explanation below). Thus, both the arrayed and the pooled RNAi library screening procedures are able to identify specific host factors for viral replication. (A list of all candidate genes from each screen and overlapping genes from respective screens is available upon request).

**Table 3:** **Gene numbers of overlapping hits identified by two independent RNAi screens**

	**Hao et al. (2008) [** 2 **]**	**Brass et al. (2009) [** 3 **]**	**Shapira et al. (2009) [** 4 **]**	**Kŏnig et al. (2010) [** 5 **]**	**Karlas et al. (2010) [** 6 **]**	**Su et al. (2013) [** 7 **]**	**Tran et al. (2013) [** 9 **]**
**Hao et al. (2008)** **[** 2 **]**		11	5	6	10	3	6
**Brass et al. (2009)** **[** 3 **]**			16	9	11	5	9
**Shapira et al. (2009)** **[** 4 **]**				12	12	7	24
**Kŏnig et al. (2010)** **[** 5 **]**					25	7	7
**Karlas et al. (2010)** **[** 6 **]**						6	4
**Su et al. (2013)** **[** 7 **]**							4
**Tran et al. (2013)** **[** 9 **]**							

### Differences between arrayed RNAi screening and pooled RNAi screen/selection

So far, most of the reported studies have used an arrayed siRNA library. An assay with quantitative characteristics, such as reporter assay or detecting viral protein expression, is a common strategy for identification of host factors using the arrayed RNAi screens. After silencing by the arrayed siRNA, the reduction of reporter activity (or the decrease in viral protein expression) reveals the host factor(s) essential for supporting influenza virus replication.

Although several virus replication-related host factors have been successfully identified by the genome-wide arrayed RNAi screens, the high-cost of the siRNA library and the requirement for a robotic liquid handling system for high throughput screening are two major drawbacks of this approach. In addition, the duration of gene silencing by siRNA transfection usually lasts for less than 1 week, which limits the usage of siRNA-based screen if long-term knockdown is needed. The lentivirus-based arrayed RNAi screen or pooled RNAi screen, on the other hand, can overcome the short-term effect of siRNA to discover host factors crucial for influenza virus replication. However, the much higher cost (compared to an siRNA library) and the requirement for a robotic liquid handling system for genome-wide arrayed shRNA screen are again major concerns. On the other hand, the cost of the pooled RNAi reagent is much lower and screen/selection can be conducted in most laboratories.

We performed a genome-wide pooled RNAi screen and identified host factors using a survival-dependent screening strategy [7]. Influenza virus infection induces cell apoptosis in certain cell types [15-17], giving the theoretical basis for our positive selection strategy for the pooled RNAi screen. Taking advantage of inhibition of the lytic cycle of influenza virus by RNAi, we selected the surviving cells with specific genes knocked down by RNAi and identified the essential host factors required for influenza virus replication. In theory, using survival rate as a phenotypic change should be able to be applied to an arrayed siRNA screen; however, the window of opportunity for measurement is narrow making it difficult to determine hits. On the contrary, the genome-integration property of lentivirus-based shRNA expression maintains host gene knockdown over a long period of time; thus, there is plenty of time to select cells refractory to the cytopathic effect of influenza infection. As a consequence, the hits identified by pooled RNAi screen may be different from those obtained by arrayed siRNA screen. Points to consider when evaluating the pooled screen versus the arrayed screen include: (i) the negative factors for influenza replication, as well as anti-apoptotic factors for cell signaling cannot be selected by pooled RNAi screen/selection due to accelerated removal of virus-infected cells, while both positive and negative factors can be identified by arrayed siRNA screen; (ii) host factors essential for cell survival or cell proliferation required for influenza replication are excluded from the list of hits during long-term gene silencing; accordingly, the hits (genes) identified by pool selection are potentially more suitable as drug targets because they are required for influenza replication without affecting host cell viability; (iii) conversely, the lentivirus-based RNAi screen provides an advantage in identification of host factors/proteins with long half-life, which might not be identified by siRNA transient transfection approach; (iv) cellular apoptotic factors would be easily selected out during positive selection since knockdown of these apoptotic genes will make cells more resistant to IAV-induced apoptosis; some of the selected cellular apoptotic factors may not be directly involved in influenza replication. In conclusion, performing screens using different approaches, such as various detection methods/parameters (i.e., RNA replication versus whole viral life cycle) and RNAi reagents (i.e., siRNA versus shRNA, or different siRNA libraries), should increase the chance of discovering essential host factors. Therefore, the gene lists from different screens should be able to complement each other.

The optimal concentration of siRNA used for gene silencing varies (1–30 nM) from study to study, and is highly dependent on the cell type and its target. Transfection of siRNA by using improper concentrations of siRNAs may result in unwanted off-target effects or incomplete gene silencing during arrayed siRNA screens, which may produce misleading results. It may be difficult to determine an optimal siRNA concentration for performing an arrayed siRNA screen. On the contrary, pooled shRNA screens take advantage of lentiviral transduction at low MOI (usually between 0.1–0.3) to deliver single copy of shRNA into cells. This low MOI remarkably reduces the probability of off-target effects. However, heterogeneity of functional siRNAs may be generated by improper processing of a shRNA hairpin, thus causing off-target effects. To minimize the off-target effects in both cases, targeted genes knocked down by at least 2 unique siRNAs (or shRNAs), which target different regions within the same gene transcript are recommended as the selection criteria, since the probability of off-target effects triggered by 2 independent siRNAs (or shRNAs) to the same target gene is extremely low. Unfortunately, the knockdown efficiency of individual siRNA in a given arrayed smart pool RNAi library is usually not known although the manufacturer guarantees that at least one of the provided siRNAs has good knockdown efficiency of 70% or more. Such knockdown information is less informative for researchers who intend to minimize the off-target effects by using two good individual siRNAs. By contrast, more than 40% of the shRNAs from the TRC RNAi library have been validated and the knockdown information of these shRNAs would provide great advantage for analysis of primary hits.

### Other factors involved in genome-wide pooled shRNA screen/selection

Genome-wide pooled shRNA screens are technically complicated, and numerous factors directly or indirectly affect the efficiency and accuracy of the screening results. Several experimental details have to be taken into consideration when performing a large-scale pooled RNAi screen. First, individual shRNA (shRNA-expressing lentivirus) should be enlarged to more than 250 representatives to guarantee that the effective shRNA(s) is not lost in subsequent experimental procedures and to compensate for the low expression level resulting from random integration sites of the shRNA expression cassette [18]. Second, an MOI of shRNA-expressing lentivirus at 0.1-0.3 is recommended to ensure that most of the surviving cells under puromycin selection receive only one copy of shRNA (one lentivirus) [18]. Reducing the MOI is not recommended, as further reduction of MOI only slightly reduces the probability of a cell being infected by two or more viruses, but significantly increases the number of cells needed for transducing the pooled shRNA lentiviruses (our unpublished data).

**Figure 1:**
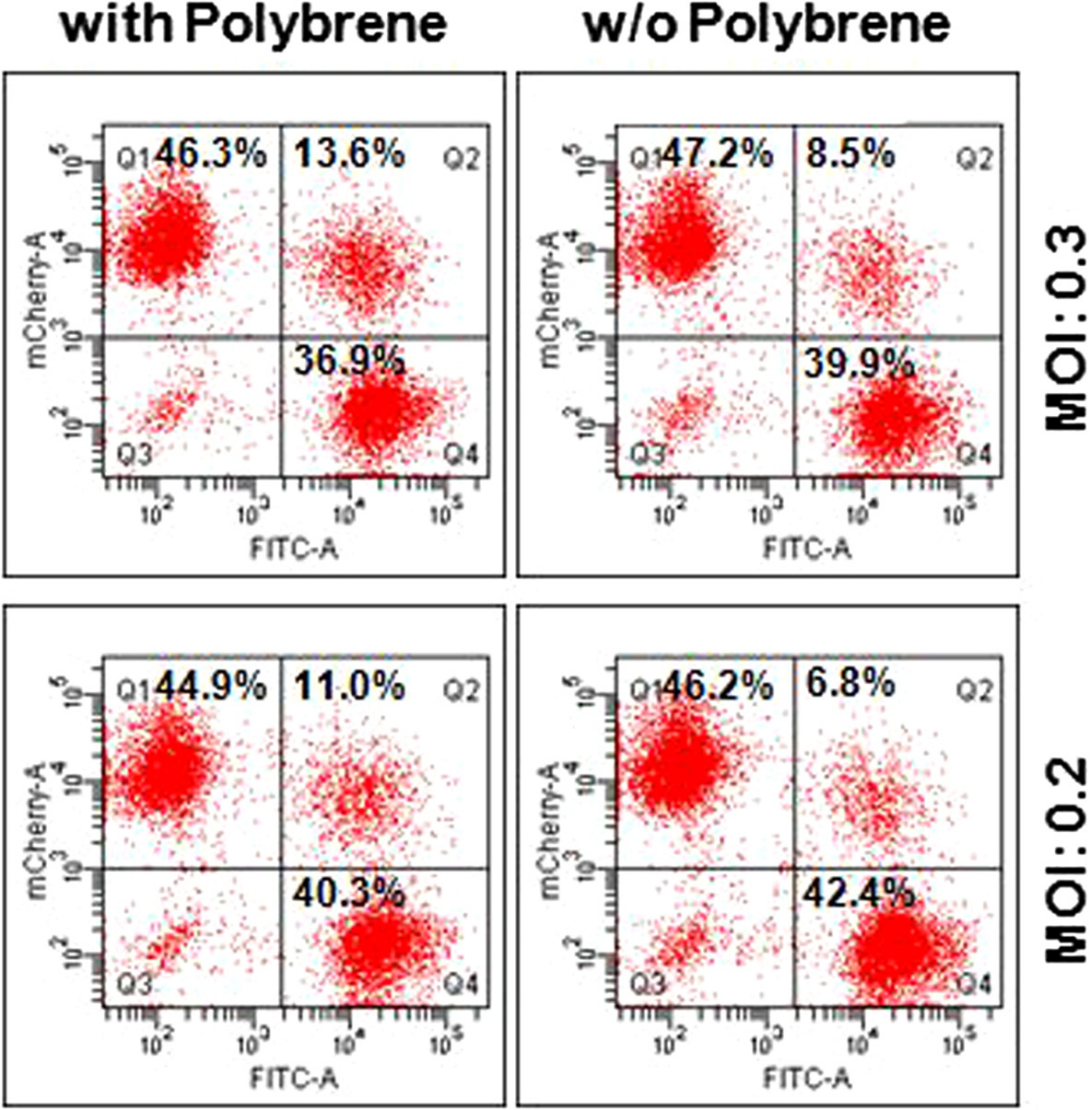
**Effect of polybrene in multiple virus infection.** A549 cells were transduced with a 1:1 ratio of lentiviruses carrying EGFP or mCherry fluorescent gene. Lentivirus infection was performed by spin infection with centrifugation at 1100 × g for 30 min in the presence or absence of 8 μg/ml polybrene (MOI = 0.2 or 0.3). Twenty four hours later, the culture medium was replaced with fresh F-12 K medium with 2 μg/ml puromycin. After puromycin selection for 5 days, the proportion of cells with EGFP(+)/mCherry(−), EGFP(+)/mCherry(+) and EGFP (−)/mCherry(+) fluore-scence signals were analyzed by flow cytometry.

Third, when performing a pooled RNAi screen, the use of polybrene (hexadimethrine bromide) during lentivirus transduction should be avoided. Polybrene is a common polycation that increases the infectivity of lentivirus 3–5 fold (our unpublished data). The cationic polymer enhances virus adsorption and transduction by neutralizing the charge between viral envelope and cellular membrane [19]. Moreover, polybrene has the potential to facilitate virus aggregation which increases the possibility of multiple virus infection during the pooled RNAi screen. By using lentivirus carrying EGFP fluorescence or mCherry fluorescence, we mimicked a pooled screening and showed that the proportions of EGFP(+)/mCherry(+) cells in the presence or absence of polybrene were 13.6% and 8.5%, respectively (Figure 1, MOI = 0.3). Assuming that the probability of cells infected by multiple lentiviruses expressing EGFP or mCherry alone is, at most, half the value of EGFP/mCherry, the probability of multiple infections in the whole population would be equal or less than 27.2% and 17% in the presence or absence of polybrene, respectively. It should also be noted that the theoretical value (calculated by the Poisson distribution equation) of multiple virus infections in the condition of MOI 0.3 is 14.3%, which is very close to the actual proportion of multiple virus infection (17%) in performing a pooled lentivirus infection without polybrene. This result demonstrates that the use of polybrene augments the proportion of multiple virus infection by at least 1.5 fold, suggesting that the use of polybrene in the pooled RNAi screen may cause multiple virus infection and thus increase the noise of hits.

Unlike the arrayed RNAi screen, the potential hits (the content of shRNAs) selected/enriched by a pooled RNAi screen must be further identified by other methods, such as barcode microarray or next generation sequencing (NGS). The barcode microarray-based method employs PCR amplification of shRNA template sequence pools, labeling of fluorophore, and hybridization to complementary DNA microarray chip(s) from an experimental group as well as a reference group. After hybridization, the fluorescent signal intensity reflects the abundance of cells expressing a certain shRNA under test conditions as compared to the reference. However, the dissimilar amplification resulting from self-annealing of hairpin sequences of shRNA may cause detection bias during microarray analysis. The external barcode tags help to optimize hybridization conditions for each probe and avoid technique bias of PCR amplification caused by the secondary structures of the shRNA template sequence. Artifacts caused by cross-hybridization, also known as unspecific probe-target interaction, are the major concern of barcode microarray hybridization in identification of target shRNA from genome-wide pooled RNAi screen. NGS or namely deep sequencing technology provides a new approach for the analysis of the hits selected by pooled RNAi screens. In brief, the experimental procedure of NGS consists of: (i) isolation of genomic DNA; (ii) PCR amplification of shRNA; (iii) restriction enzyme digestion and gel purification of the PCR products; and (iv) ligation of PCR products to an adaptor for subsequent NGS analysis. In general, maintaining the fidelity of shRNA population during preparation of PCR fragments for NGS is important for subsequent hit determination. Our recommendations for preparing shRNA-containing PCR fragments for NGS are as follows: (i) Prepare appropriate amounts of genomic DNA without loss of shRNA complexity. Low amounts of genomic DNA may not cover the whole population of shRNA in a typically genome-wide-scale negative selection experiment; (ii) Avoid creating conditions that generate sequencing bias during PCR amplification, such as over-amplification and two-rounds of PCR. Exponential PCR cycles without generating heteroduplex that are derived from annealing of two shRNA sequences occurring at the later stage of PCR are recommended; (iii) Prepare PCR fragments with half-arm of shRNA cassette for NGS, if possible, because full-length shRNA may impede sequencing; (iv) Templates should contain a 5' end phosphate and a 3' end hydroxyl group for efficiently adding adaptor for NGS; (v) Purify restricted PCR fragments by gel electrophoresis for deep sequencing. A protocol for genome-wide pooled RNAi screen can be found at: http://rnai.genmed.sinica.edu.tw/file/protocol/PooledScreen_SequencingProtocol.pdf.

By detection of the amounts of the shRNA sequence, the NGS approach may hold greater promise for accuracy than that of the barcode microarray-based method for deconvolution of genome-wide pooled RNAi screening output. There are at least four advantages of using the NGS method in place of the barcode microarray hybridization. First, NGS technology is a cost-effective approach because it measures the presence of large quantities of distinct shRNA sequences in a short time. Secondly, NGS greatly improves the sensitivity and efficacy of detection by offering a digital readout of even very small amounts of shRNA species. Third, the NGS method provides a greater detection range and better resolution of measurements, which enables clear discrimination of true hits from background noise. Finally, NGS is a more flexible approach for the identification of hits from a genome-wide RNAi screen, which can be easily incorporated into any high-complexity shRNA library without generating new information of barcode and linkage to individual shRNA sequence.

### Conclusions and perspectives

Genome-wide RNAi screen is a useful tool for studying the gene functions by manipulation of gene expression. Both arrayed RNAi screen and pooled RNAi screen have been successfully conducted to identify host factors crucial for influenza virus replication. Two previous influenza screening reviews focusing on arrayed RNAi screens have uncovered host factors and cellular networks involved in the influenza virus replication [20,21]. Here, we review the current genome-wide influenza screens; both arrayed RNAi screens and pooled RNAi screens. A comparison of the published genome-wide RNAi screens of influenza research (2008–2013) revealed that only a limited number of overlapping hits were identified among these RNAi screens, and such an inconsistency is very likely due to the different screening approaches/ methods used. Overall, there was zero commonality among the seven different RNAi screens to identify host factors for influenza virus infection. This result is consistent with previous genome-wide RNAi screens that searched for host targets to combat HIV infection (2008–2011), in which there was zero gene commonality across the board [22-27]. Although overlapping genes were found among some of the screens, the reason why an essential host gene identified in one screen could not be fully reproduced in the others remains unknown. It is very likely because different experimental parameters were used in each screen, which may directly or indirectly affect the efficiency and accuracy of the RNAi-based screening. Moreover, the analyzed time points covered by the different strategies of RNAi screens, e.g., short-term knockdown by siRNA or long-term knockdown by lentivirus-based shRNA, also influence the populations of host factors identified by the RNAi screens. Other factors, e.g., the genes involved in shRNA processing, may also result in the biased selection of candidate genes in different RNAi screens. As a consequence, the different features of the arrayed siRNA screen and the pooled shRNA screen should be taken into consideration while designing a feasible genome-wide RNAi screen procedure. Here, we have shown several benefits as well as tips in performing genome-wide pooled shRNA screens. Although there are still some concerns about pooled shRNA screens, these concerns can be technologically overcome through careful design of the screening procedures, use of proper controls and stringent standards for identification of the hits. In combination with the advances in NGS technology, the genome-wide pooled shRNA screen can thus be hailed as the second genomics wave.

## Abbreviations

RNAi: RNA interference
IAV: Influenza A virus
HA: Glycoproteins hemagglutinin
NA: Neuraminidase
M1: Matrix protein
M2: Ion channel protein
NP: nucleoprotein
vRNP: Viral ribonucleoprotein
vRNA: Viral RNA
RdRp: RNA-dependent RNA polymerase
VSV-G: Vesicular stomatitis virus glycoprotein G
TRC: The RNAi consortium
NGS: Next generation sequencing
